# Case of Sanguineous Apoplexy Occurring in a Child

**Published:** 1822-03

**Authors:** T. H. Greenhow


### ART. II.—*Case of Sanguineous Apoplexy occurring in a Child.* By T. H. GREENHOW, Esq.

FLORA THOMAS, aged two years and a half, of a scrofulous constitution, who had always been fed upon very poor diet, consisting principally of oatmeal, had been, for many months, subject to great functional disorder of the abdominal viscera. The abdomen was much enlarged, hard, and tense; the bowels irregular, requiring the frequent use of medicine to procure evacuations, which were always chapped, green, and slimy. She had grinding of the teeth and much starting during sleep, picking of the nose, and other marks of abdominal irritation, with occasional attacks of fever; for which complaints frequent doses of calomel, and other purges, were prescribed; and now and then a glyster, containing oleum terebinthinæ, was administered. Under this treatment, the abdomen became softer and less tumid, and the stools more natural and regular.

The child's mother had been several times alarmed by its being seized with a sudden gasping for breath, becoming, to use her own words, quite purple in the face. After continuing for a minute or two, this gradually disappeared, and the child breathed again with freedom. I had never been present during one of these attacks: but, on the 11th of January, I received a hasty summons to visit her, the messenger stating that she was dying. On my arrival, I found her quite dead, her face being dark and livid. The attack had taken place about ten minutes before I got there, precisely in the way before described: the child's face was convulsed, it seemed gasping for breath, struggled for a minute or two, and then became quite motionless. With the assistance of another practitioner, who had also been sent for during the alarm, I opened the external jugular vein: nearly three ounces of blood flowed from it, which led us to hope the child might still be restored. It was immediately plunged into a warm bath, the lungs were inflated, and other appropriate means used; but without success.

The following morning, with the assistance of the gentleman above mentioned, I examined the body. A large quantity of blood was found poured out upon the surface of the brain, and the vessels were all much distended; but the substance of the brain itself was without disease. The quantity of blood was not measured; but, in removing the calvaria, which required considerable force, many ounces escaped into a basin, which was placed to receive it. The lungs were in some parts gorged with blood, being dense, dark, and liver-like. The heart was exceedingly small, and completely empty. No water was contained in the chest or pericardium. All the abdominal viscera were greatly enlarged, particularly the liver, which was truly immense, and very firm, though apparently not essentially altered in its texture. The gall-bladder contained no bile. The spleen and kidneys were unusually large; and many of the mesenteric glands showed evident marks of disease, being much enlarged and indurated.

From the above appearances I am led to conclude, that the apoplectic attack, which caused the death of this child, was occasioned by the pressure of the enlarged viscera of the abdomen upon the great blood-vessels preventing the reflux of the blood to the heart.

*Newcastle; January 15th,* 1822.

